# Response to a wild poliovirus type 2 (WPV2)-shedding event following accidental exposure to WPV2, the Netherlands, April 2017

**DOI:** 10.2807/1560-7917.ES.2017.22.21.30542

**Published:** 2017-05-25

**Authors:** Erwin Duizer, Wilhelmina LM Ruijs, Charlie P van der Weijden, Aura Timen

**Affiliations:** 1 Centre for Infectious Diseases Control, National Institute for Public Health and the Environment (RIVM), Bilthoven, the Netherlands; 2 Municipal Health Service Flevoland, Lelystad, the Netherlands

**Keywords:** poliovirus, wild poliovirus type 2 (WPV2), containment, GAPIII, hygiene measures

## Abstract

On 3 April 2017, a wild poliovirus type 2 (WPV2) spill occurred in a Dutch vaccine manufacturing plant. Two fully vaccinated operators with risk of exposure were advised on stringent personal hygiene and were monitored for virus shedding. Poliovirus (WPV2-MEF1) was detected in the stool of one, 4 days after exposure, later also in sewage samples. The operator was isolated at home and followed up until shedding stopped 29 days after exposure. No further transmission was detected.

The Dutch National Polio Laboratory of the National Institute of Public Health and the Environment (RIVM) was informed about a partly aerosolised high titre spill of monovalent wild poliovirus type 2 (WPV2-MEF1) in a vaccine manufacturing plant in the centre of the Netherlands on 3 April 2017. Following the World Health Organization (WHO) recommendations [1], all staff working with infectious polioviruses have to be vaccinated, however, vaccines (inactivated polio vaccine (IPV) and oral polio vaccine (OPV)) protect against disease, not against infection. Therefore, all staff who might have been exposed needed to be followed up to check for infection and excretion of the virus. Two fully vaccinated operators with possible exposure related to the event were identified and monitored. Immediately after the detection of poliovirus in the stool of the one of them on 7 April, the Centre for Infectious Disease Control (CIb) of the National Institute for Public Health and the Environment (RIVM) formed an outbreak management team to closely monitor the situation, and to enforce and facilitate stringent hygiene measures and voluntary home isolation. Here we describe the response to the event including considerations and re-adjustments of follow-up measures, according to newly available information.

## Containment and monitoring

### Exposed operators

Following the possible exposure, the two fully vaccinated operators were monitored according to the protocol of the facility. Advice on stringent personal hygiene was given on the day of the incident, as well as instructions to avoid contact with unvaccinated persons. Throat swabs and stool specimens were collected on day 3/4 and day 7/8 after exposure. On Friday, 7 April, (day 4 after exposure), throat and stool samples were collected from both operators and these were analysed by RT-PCR. The faecal sample of one of the exposed operators was positive for poliovirus by RT-PCR. On Sunday, 9 April, virus cultures on L20B and rhabdomyosarcoma (RD) cells were also positive and the samples were processed for full poliovirus VP1 sequencing. On 10 April, sequencing of this stool sample showed 100% identity of full VP1 to WPV2 (MEF-1 IPV strain) (data not shown). On the same day, this confirmation of a WPV2 infection in an operator in the Netherlands was reported to the WHO, according to the International Health Regulations [2]. The European Commission and relevant authorities in the European Union (EU) Member States were informed through the EU Early Warning and Response System (EWRS).

Throat swabs of the infected operator collected on day 4 and 8, as well as all samples of the second operator, remained negative in RT-PCR on clinical material and in virus isolation.

### Diagnostic procedures and laboratory containment

Samples received on 7 April were processed in the National Polio Laboratory at the RIVM under poliovirus-containment-approved biosafety level (BSL)-2   conditions. From 11 April, all samples of the infected operator were processed under BSL-3 containment, including the use of filtering face piece (FFP)3 masks and excluding the presence of other staff. All other samples (from contacts and sewage) were initially processed under BSL-2 conditions. As soon as cultures started showing cytopathogenic effects (CPE) indicative of virus propagation, the closed culture tubes were transported to the BSL-3 laboratory.

All samples in this monitoring programme were analysed by RT-PCR for generic enterovirus and poliovirus detection and specific WPV2 detection and all samples were processed for virus isolation according to the WHO protocol [3]. The cultures of the infected operator were disposed of (following BSL-3 waste management guidelines) as soon as CPE appeared and the WPV2 PCR on the stool suspension was positive, except for six samples. For these six samples, the L20B cultures were opened and viral RNA was extracted for sequencing. The sewage samples were processed as described previously [4] under BSL-2 containment. All laboratory staff who processed WPV2-positive materials under BSL-2 containment were followed up by day 3/4 and day 7/8 stool sample analysis. All remained negative for WPV2 excretion.

### Follow-up of the infected operator

The infected operator was followed up by daily stool sampling. This was continued until the stool tested negative for at least 3 consecutive days, which is in line with the WHO ‘Global Action Plan to minimize poliovirus facility-associated risk after type-specific eradication of wild polioviruses and sequential cessation of oral polio vaccine use’ (GAP III) [1].

Starting with 7 April, the infected operator was signed off from work and in voluntary home isolation under daily supervision of the local public health service. The infected operator resides in an area of the Netherlands with high vaccination coverage, which does not belong to the so-called Bible belt, an area where some inhabitants object to vaccination on religious grounds and that has a lower coverage [5,6]. Until 13 April, the infected operator used the sole toilet in their apartment together with two household members. The infected operator was instructed by the local public health service to follow strict hygiene measures such as flushing with toilet lid closed, disinfection with chlorine after every defecation and strict hand hygiene (i.e. using medical gloves while using the toilet, sampling and disposing of faeces, and washing hands afterwards). Starting with 14 April, all stools from the infected operator were collected in a disposable system as the one recommended for Ebola virus disease patients [7]: a toilet chair, a plastic bag with absorbent material, in a plastic waste container. These disposable materials were disposed of in a high-level containment box before transportation to and immediate destruction at the designated waste incineration plant. The infected operator’s stools tested positive until 29 April and once again on 1 May (Figure). They were in home isolation for 32 days.

**Figure:**
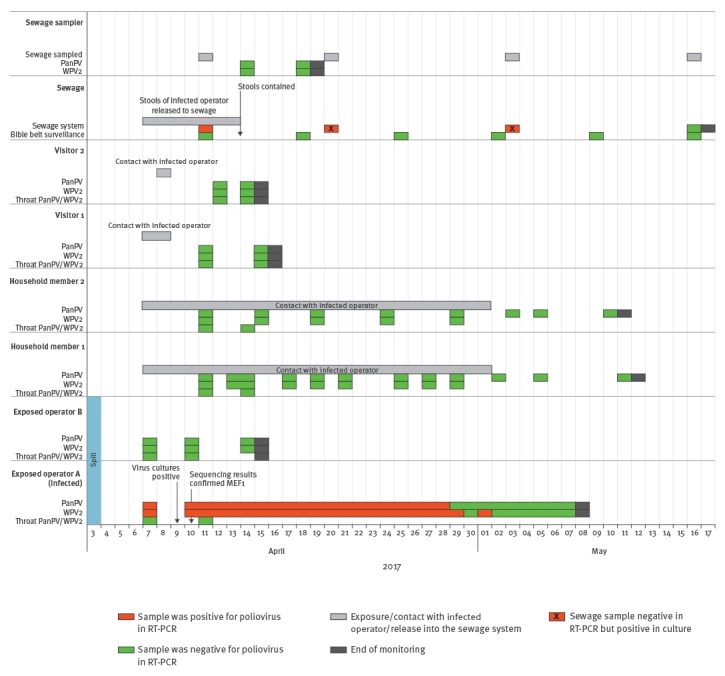
Timeline of the response, including sampling dates and poliovirus detections, to wild poliovirus type 2 (WPV2)-shedding event following accidental exposure to WPV2, the Netherlands, April 2017

Sewage sampler is the sampler of the sewage system downstream of the infected operator’s residence.

Sewage system downstream of the infected operator’s residence.

### Follow-up of household contacts

An investigation of the contacts was undertaken on 8 April, revealing four persons who had been in the house of the infected operator in the previous days (two visitors and two household contacts). All were fully vaccinated with IPV according to the national immunisation schedule. The two visitors visited the infected operator on 7 and 8 April (one of them visited on both days); both were monitored by stool and throat samples collected on day 4 and day 6/8 after contact. All samples of these two contacts were negative for poliovirus and monitoring was stopped (Figure).

The household members were followed up by throat swabs and stool sampling starting with day 3/4 following the first possible exposure (Figure). Monitoring of the two household members was continued until 10 days after the end of virus shedding by the infected operator. As at 12 May, all samples were negative by RT-PCR and monitoring was stopped.

Advice on personal hygiene was provided to all contacts as well as instructions to avoid contact with unvaccinated persons, and to refrain from visiting regions with low vaccination coverage and from specific activities such as swimming. The two household members were explicitly requested not to defecate outside their home. Apart from the two visitors and the two household members, no visitors were allowed to enter the home of the infected operator while they were shedding virus.

### Environmental surveillance

During the first week after exposure, the toilet in the household of the infected operator was not disconnected from the sewage system and the WPV2 contaminated stool was thus not contained. To monitor WPV2 and its disappearance from the sewage system downstream of the residence, the system was sampled on 11 and 20 April and again on 3 and 16 May. WPV2 was detected by direct RT-PCR and by virus isolation in one of two sewage samples collected on 11 April. In two of five sewage samples collected on 20 April, and one of two samples collected on 3 May, WPV2 was detected only after virus isolation. Both samples collected on 16 May were negative for WPV2 and sewage monitoring downstream of the residence of the infected operator, was stopped.

The individual who collected the samples on 11 April was followed up by stool testing on day 4 and 7 after sampling: both stool samples were negative for poliovirus by RT-PCR and virus isolation. The individuals who collected later sewage samples were not followed up since WPV2 could not be detected in the concentrated samples by RT-PCR and it was either not detected or only after culture.

As a precautionary measure and to allow for early detection, the routine environmental surveillance applied in the regions with lower vaccination coverage (Bible belt) [4] was increased to weekly sampling from 11 April and was continued up to 16 May. So far, all samples from the routine environmental surveillance have tested negative for poliovirus.

## Background

In 2015, WPV2 was officially declared eradicated by the World Health Assembly and the GAPIII was adopted [1]. Poliovirus facilities that serve critical international functions, including IPV and Sabin-IPV production, should manage biorisk appropriately, to minimise the risk of virus reintroduction into the environment and the community, and GAPIII requires very stringent containment of all PV2-related processes. From the PV2 eradication perspective, this is reasonable, however, it does not directly relate to a concrete public health threat, especially in countries with a strong hygienic infrastructure i.e. management of sewage waste water and a full IPV vaccination programme.

## Discussion

We report on an infection with a WPV2 following a spill in a vaccine manufacturing plant. This is the first reported incident of its kind while stringent biorisk management systems in accordance with the GAPIII should be in place. Still, we identified gaps in the guidance for containment of WPV2 when an employee of such a plant excretes WPV2. The lessons learned will serve to update our national public health guidelines on poliovirus risk management.

Since the GAPIII document does not provide accurate descriptions for practical implementation of containment requirements, and the Dutch guidelines for polio were not GAPIII-compliant, we opted to combine a proportional public health response and several GAPIII requirements to deal with this public health incident.

The current follow-up protocol for possibly exposed employees of a facility requires the first sampling of stool and a throat swab on day 3/4 after the possible exposure. It aims at reliable exclusion of poliovirus infection and excretion based on the assumption that in the majority of cases, a spill does not result in an infection. Biorisk management procedures for WPV2 incidents in the post-eradication period, require early detection of poliovirus excretion. In order to achieve results timely, for WPV2 incidents, stool samples should be collected daily, starting immediately after the incident and be analysed by real-time RT-PCR. Even if direct detection by PCR may be less sensitive than virus isolation from cell cultures, the detection limits of current optimised protocols (< 500 poliovirus RNA per gram stool) are such that virus loads below the detection limit for PCR are unlikely to pose a real risk for ongoing transmission.

Immediately upon the confirmation of WPV2 shedding by one of the operators, according to the Dutch procedures for outbreak control, an outbreak management team was convened. In our experience, multidisciplinary expertise is needed for a detailed risk assessment and effective incident management [8]. We focused in our risk assessment and containment measures on the faecal-oral transmission route, as the throat swabs of the infected operator were negative. These negative results are in agreement with the literature that shows that oral virus excretion by fully vaccinated persons is highly unlikely [9,10].

Initially, the WPV2-contaminated stools of the infected operator were not contained. The risk of poliovirus transmission through the sewage system was estimated to be very small since the sewage system concerned was a closed system with dilution and treatment at the sewage treatment plant (STP). Furthermore, it was checked and confirmed that there was hardly any contact between staff and potentially WPV2-contaminated sludge, the sewage sludge was incinerated and all workers of the STP were fully vaccinated against polio. The local public health services ensured that no sewage system maintenance works were in progress or planned in the near future downstream of the infected operator’s residence up to the STP. One sewage pit downstream of the infected operator’s residence remained positive for WPV2 for at least 20 days after disconnection from the sewage system, showing the need for containment of WPV2-positive stools. Disappearance of infectious virus from the local sewage system is the result of flushing of pipes, dilution and inactivation, and depends on factors such as number of households discharging to the system, constitution of waste discharged, precipitation, structure and integrity of the sewage system and temperature. As we show, and others suggested, this may take weeks to months (this study and [11]).

The containment of the stools of the infected operator was a challenge because a standard chemical toilet does not inactivate poliovirus and admittance to a hospital would have increased the risk of transmission of poliovirus to immunocompromised patients. Finally, we found a disposable system as used for Ebola virus disease patients to be useful and easily applicable at the home of the infected operator [7].

Balancing personal needs and the risk of infection, two family members of the infected operator remained in the same household throughout the isolation period. They were monitored and did not excrete poliovirus; they also agreed to comply with stringent hand and toilet hygiene and considerable restriction of their contacts and freedom to move outside the house. From a biorisk management perspective, this situation was far from ideal, however, isolation of the infected employee in a specially contained facility for more than 4 weeks would have severely disrupted family life.

## Conclusions

Retrospectively, the measures undertaken to manage the public health risk were appropriate and proportional: during comprehensive monitoring no further spread of the virus was detected and there was acceptable impairment in the family life of the infected operator. The infected operator complied well with the stringent hygiene measures and prevented transmission of WPV2 to his household contacts. However, the containment of WPV2 in this event was not according to the biorisk management level expected in GAPIII. The GAPIII document is overall clear on what level of biorisk management for WPV2 is expected to prevent reintroduction of any PV2 into the environment and the community. It does, however, not provide practical guidance on how this should be achieved or on how to deal with an infected employee in the community (this report) or in a clinical/hospital setting [12]. The reported incident leading to WPV2 shedding by an exposed operator highlights that pre-GAPIII procedures and guidelines are insufficient in the post-GAPIII era. The biorisk management requirements as formulated in GAPIII need to be further translated into practical, probably country-specific, guidelines.
